# Primary Hyperparathyroidism Influences the Expression of Inflammatory and Metabolic Genes in Adipose Tissue

**DOI:** 10.1371/journal.pone.0020481

**Published:** 2011-06-17

**Authors:** Monika H. E. Christensen, Simon N. Dankel, Yngve Nordbø, Jan Erik Varhaug, Bjørg Almås, Ernst A. Lien, Gunnar Mellgren

**Affiliations:** 1 Institute of Medicine, University of Bergen, Bergen, Norway; 2 Hormone Laboratory, Haukeland University Hospital, Bergen, Norway; 3 Department of Surgery, Haukeland University Hospital, Bergen, Norway; 4 Department of Surgical Science, University of Bergen, Bergen, Norway; Governmental Technical Research Centre of Finland, Finland

## Abstract

**Background:**

Primary hyperparathyroidism (PHPT) is characterised by increased production of parathyroid hormone (PTH) resulting in elevated serum calcium levels. The influence on bone metabolism with altered bone resorption is the most studied clinical condition in PHPT. In addition to this, patients with PHPT are at increased risk of non-skeletal diseases, such as impaired insulin sensitivity, arterial hypertension and increased risk of death by cardiovascular diseases (CVD), possibly mediated by a chronic low-grade inflammation. The aim of this study was to investigate whether adipose tissue reflects the low-grade inflammation observed in PHPT patients.

**Methodology/Principal Findings:**

Subcutaneous fat tissue from the neck was sampled from 16 non-obese patients with PHPT and from 16 patients operated for benign thyroid diseases, serving as weight-matched controls. RNA was extracted and global gene expression was analysed with Illumina BeadArray Technology. We found 608 differentially expressed genes (q-value<0.05), of which 347 were up-regulated and 261 were down-regulated. Gene ontology analysis showed that PHPT patients expressed increased levels of genes involved in immunity and defense (e.g. matrix metallopeptidase 9, S100 calcium binding protein A8 and A9, CD14, folate receptor 2), and reduced levels of genes involved in metabolic processes. Analysis of transcription factor binding sites present in the differentially expressed genes corroborated the up-regulation of inflammatory processes.

**Conclusions/Significance:**

Our findings demonstrate that PHPT strongly influences gene regulation in fat tissue, which may result in altered adipose tissue function and release of pathogenic factors that increase the risk of CVD.

## Introduction

Primary hyperparathyroidism (PHPT) is one of the most common endocrine disorders [1]. Worldwide PHPT is most often seen in postmenopausal women [2] and in Scandinavia the prevalence was found to be higher than 2% in elderly women [3], [4]. The diagnosis of PHPT is biochemically determined by increased serum parathyroid hormone (PTH) levels leading to increased concentrations of serum calcium. 85% of the cases of PHPT are caused by a single, benign parathyroid adenoma. Parathyroidectomy cures 90–95% of these patients, measured by normalisation of PTH and calcium levels [5]. Elevated PTH levels exert a well-described effect on bone metabolism, leading to increased bone turnover and osteoporosis. Parathyroidectomy reduces markers of bone turnover and increases bone density [6]. In the last decades there has been a shift in the clinical findings in patients with PHPT. Due to improved diagnostic procedures and analytical methods, mild PHPT is now often discovered in routine health controls. Classical findings such as skeletal lesions and nephrolitiasis have become rare.

Metabolic changes observed in patients with PHPT include impaired insulin sensitivity, elevated LDL-cholesterol, decreased HDL-cholesterol, and elevated circulating inflammatory markers such as high-sensitive C-reactive protein and tumor necrosis factor-alpha [7]. An increased risk of cardiovascular diseases (CVD) in patients with PHPT has been reported [8]. In line with this, patients with PHPT were shown to have an increased risk of hypertension [9], [10] and impaired glucose tolerance [11]. CVD seems to be more evident in patients with severe PHPT, but also PTH levels within the upper part of the normal range is associated with an increased incidence of CVD [12]. Chronic low-grade inflammation in PHPT may play an important role in the development of CVD in these patients, since inflammation is a key component in the pathogenesis of atherosclerosis [13].

Adipose tissue is an important endocrine organ with crucial functions in the regulation of energy homeostasis, insulin sensitivity, and lipid and carbohydrate metabolism [14]. Others and we have described that adipose tissue in obesity shows a marked increase in the expression of inflammatory genes and release of adipocytokines [15], [16]. To our knowledge the function of adipose tissue has not been described in patients with PHPT. Through global gene expression profiling we identified potential risk genes with differential expression in subcutaneous adipose tissue of non-obese PHPT patients compared to a weight-matched control group. The most pronounced finding was an up-regulation of inflammatory genes in PHPT patients while genes with functions in fatty acid and carbohydrate metabolism were down-regulated.

## Materials and Methods

### Ethics statement

The study war performed according to the principles expressed in the declaration of Helsinki and all enrolled subjects signed an informed written consent. The Western Norway Regional Committee for Medical Research Ethics (REK) approved the study.

### Subjects and study design

The study included 16 patients (14 females and 2 males) undergoing surgery for PHPT and 16 control subjects (11 females and 5 males) operated for benign thyroid hypertrophy. Subjects were recruited in the period from September 2007 to September 2009. Subcutaneous adipose tissue was obtained from the neck at the beginning of the surgery. All patients were operated at the Department of Endocrine Surgery, Haukeland University Hospital, Bergen, Norway. The diagnosis of hyperparathyroidism was based on elevated serum PTH (ref. range: 1.3–6.8 pmol/L) and ionised calcium levels (ref. range: 1.13–1.28 mmol/L). Exclusion criteria were body-mass index (BMI)>29 kg/m^2^ and any kind of known systemic inflammatory disease, such as inflammatory bowl disease, rheumatological diseases and chronic obstructive lung disease. Weight, height and medical history were recorded before surgery.

### Biochemical analysis

Blood samples were drawn the day before surgery from all PHPT patients and within the first day after operation from the control subjects. Ionised calcium, phosphate, creatinine, total cholesterol, HDL-cholesterol and LDL-cholesterol were analysed immediately by standard laboratory methods. PTH was measured with a two-site chemiluminescent immunometric assay for intact PTH (Immulite 2000, Siemens, UK). The inter-assay variation was 6.3% at a concentration of 5.6 pmol/L and 8.8% at 40 pmol/L.

### Homogenisation and RNA extraction

Biopsies of subcutaneous adipose tissue obtained during surgery were immediately frozen and stored in liquid nitrogen until homogenisation and RNA extraction. Frozen adipose tissue was transferred into 2 ml safe-lock eppendorf tubes with rounded bottom. A 5 mm metal bead (Millipore, USA) and one ml quiazol lysing buffer (Qiagen, Germany) were added and homogenisation in a TissueLyser (Qiagen) followed immediately. RNA extraction was performed using the RNeasy Lipid Tissue Midi Kit (Qiagen). Samples were treated with the RNase-Free DNase Set and the RNeasy MiniElute Cleanup Kit (Qiagen). Amount and quality of the extracted RNA were measured by the NanoDrop® ND-100 spectrophotometer (NanoDrop Technologies, USA) and the Agilent 2100 Bioanalyzer (Agilent Technologies, USA).

### Microarray analysis

Microarray analysis was performed using the Illumina HumanRef-8 v.3 Expression BeadChips, which targets about 24,500 annotated RefSeq transcripts and covers 18,631 unique curated genes. In total, 32 microarrays from 16 biological replicates, respectively 16 patients and 16 controls, were performed. 370 ng of total RNA was used for the Illumina TotalPrep Amplification Kit (version 27.07.09, Applied biosystems/Ambion, USA) to generate biotin-labelled, amplified RNA. Quality of labelled cRNA was measured using the NanoDrop® ND-100 spectrophotometer and the Agilent 2100 Bioanalyzer. 750 ng biotin-labelled cRNA was used for hybridisation to gene-specific probes on the Illumina microarrays (product number BD-102.0203) according to the Whole-Genome Gene Expression Direct Hybridization Assay Guide (Illumina Inc, Nov 2006). The Illumina arrays were then scanned with the iScan Reader, based on fluorescence detection of the biotin-labelled cRNA. The Illumina microarray data are MIAME compliant and the raw data have been deposited in the database ArrayExpress (ArrayExpress accession: E-TABM-1119). Analyses were performed at the Norwegian Microarray Consortium (NMC) Core Facility, University of Bergen, Norway.

### Microarray data extraction and analysis

Raw data were imported into the GenomeStudio Data Analysis Software and quality controls were performed. Seven different control categories were built into the Whole-Genome Gene Expression Direct Hybridization Assay system, covering every aspect of an array experiment. Looking at the technical controls in GenomeStudio, one of the samples (sample C-111, a male control) had a different distribution of signals in several control plots, such as the box plot visualising the variation within an array and between arrays, the line plot of detected genes, the line plot of noise and the line plot of labelling control across samples. All samples were included in further quality control, outlier detection, and pre-processed using the J-Express software version 2009 (MolMine, Norway) [17]. Quality control and analysis in this software was done on log2-transposed data. Correspondence Analysis [18] and hierarchical clustering with Pearson Correlation as a distance measure were used on both the un-normalised and quantile normalised dataset [19]. The Correspondence Analysis plot was used to look for greatest co-variance in the dataset. Sample C-111 was an outlier in the un-normalised dataset in the Correspondence Analysis plot, and together with the outlier detection of control probes in GenomeStudio we decided to exclude this sample before further analysis.

Significance Analysis of Microarrays (SAM) [20] was used to look for differentially expressed genes, which were defined by q-value<0.05. Protein Analysis Through Evolutionary Relationships (PANTHER) (version 6.1, http://www.pantherdb.org) was used to organise differentially expressed genes in categories representing biological processes and molecular functions. We looked for over-representation of differentially expressed genes in such categories, relative to the expected representation in the whole genome. The Bonferroni correction for multiple testing was used in the calculation of p-values for the over-represented PANTHER categories.

### Validation of microarray data by qPCR

Nine genes of interest were selected for validation of the microarray results by quantitative real time PCR (qPCR). qPCR analyses were performed on all samples included in the study. The SuperScript Vilo cDNA Synthesis Kit (Invitrogen GmbH, Germany) was used for cDNA synthesis, followed by qPCR with the LightCycler480 Probes Master kit and the LightCycler480 rapid thermal cycler system (Roche Applied Science, USA). Probes and primers for target genes (Table 1) were designed using Univeral ProbeLibrary (UPL) Assay Design Center (Roche Applied Science), software version 2.45. For genes with more than one transcript variant (CD14 and FOLR2), primers and probes were designed to cover all variants. The UPL human TATA box binding protein (TBP) Gene Assay (Roche Applied Science) was used for the reference gene. Target genes were amplified in duplex with TBP except for IL8, CD14 and SCD since duplex affected the amplification efficiency of their transcript. Instead, expression levels were calculated relative to the mean TBP concentration from four of the duplex runs. Data analysis was performed using PASW Statistics 18 for Mac and the statistical package provided in Excel (Microsoft). To assess differences between the two groups we used the Mann-Whitney U Test. All tests were two-sided and p<0.05 was considered to be statistically significant.

**Table 1:** Primers used in this study.

GeneBank ID	Genes	PCR primers (5′-3′)	Probe nr (UPL)	Product size (bp)
NM_004797.2	*AdipoQ*	AGG GTG AGA AAG GAG ATC CAG TCC TTT CCT TTG GAT T	41	113
NM_002982.3	*CCL2*	AGT CTC TGC CGC CCT TCT GTG ACT GGG GCA TTG ATT G	40	93
NM_000591.2 (tv 1) NM_0010400211 (tv 2)	*CD14*	GTT CGG AAG ACT TAT CGA CCA T ACA AGG TTC TGG CGT GGT	74	95
NM_013402.4	*FADS1*	TCT CTC CTG ATT GGA GAA CTG TG CCG GAA CTC ATC TGT CAG C	26	81
NM_004104.4	*FASN*	CAG GCA CAC ACG CTG GAC CGG AGT GAA TCT GGG TTG AT	11	92
NM_000803.4 (tv 1) NM_001113534.1 (tv 2) NM_001113536.1 (tv 3) NM_001113535.1 (tv 4)	*FOLR2*	CTA TGA GTG CTC ACC CAA CCT CCA GGA AGC GTT CTT TGC	81	74
NM_000584.2	*IL8*	AGA CAG CAG AGC ACA CAA GC ATG GTT CCT TCC GGT GGT	72	62
NM_004994.2	*MMP9*	GAA CCA ATC TCA CCG ACA GG GCC ACC CGA GTG TAA CCA TA	6	67
NM_005063.4	*SCD*	CCT AGA AGC TGA GAA ACT GGT GA ACA TCA TCA GCA AGC CAG GT	82	65

UPL, Universal ProbeLibrary probes (Roche Applies Science); tv, transcript variant.

### oPOSSUM binding site analysis

To analyse which transcription factors might regulate the differentially expressed genes, we uploaded the lists of up- and down-regulated genes to the oPOSSUM program online (http://www.cisreg.ca/cgi-bin/oPOSSUM/opossum) [21]. oPOSSUM uses a database of conserved transcription factor binding sites (TFBS), and calculates significance values (Z-score and Fisher score) for over-representation of TFBS in a list of co-expressed genes. Transcription factors for which there are more binding sites in the co-expressed genes than expected are over-represented. A list of transcription factors predicted to regulate a significant number of the co-expressed genes was retrieved. Top 10% of conserved regions, 80% matrix match threshold, Z-score>5 and Fisher score <0.05 were chosen as output parameters (vertebrate matrix).

## Results

### General data of the study population

The analysis included 31 individuals, thereof 16 PHPT patients and 15 controls. Characteristics of the study population are listed in Table 2. All patients had elevated serum PTH concentrations due to a parathyroid adenoma. Six months after surgery all patients had normalised serum PTH and ionised calcium levels. One patient had serum PTH of 3.8 pmol/L (ref. range 1.3–6.8 pmol/L). This patient had an elevated serum ionised calcium level of 1.45 mmol/L (ref. range: 1.13–1.28 mmol/L), osteoporosis and an adenoma in one of the parathyroid glands localised by ultrasonography and scintigraphy. The diagnosis of an adenoma in the parathyroid gland was histologically confirmed after extirpation of the gland. Six months after operation this patient had serum PTH of 2.8 pmol/L and serum ionised calcium was normalised at 1.24 mmol/L. All control subjects had serum PTH and calcium within the normal reference range. None of the patients or controls had known diabetes mellitus or atherosclerotic disease. Six of the patients with PHPT and one patient in the control group used medication for hypertension.

**Table 2:** Anthropometric and biochemical measurements of the PHPT patient and control groups.

	PHPT(n = 16, 14 female)	Control(n = 15, 11 female)	
	Median (IQR)	Median (IQR)	P-value
**Age** (years)	60.0 (49.3–69.2)	47.0 (38.0–56.0)	0.01
**BMI** (kg/m^2^)	25.4 (23.5–27.2)	25.0 (22.9–26.9)	0.78
**PTH** (1.3–6.8 pmol/L)	11.5 (9.6–15.6)	3.3 (2.1–4.2)	<0.001
**iCa** (1.13–1.28 mmol/L)	1.47 (1.41–1.54)	1.24 (1.21–1.25)	<0.001
**Phosphate** (0.85–1.50 mmol/L)	0.87 (0.70–1.01)	1.08 (0.91–1.23)	0.02
**ALP** (35–105 U/L)	93.0 (74.0–119)	68.0 (51.5–82.0)	0.01
**TSH** (0.4–4.5 mIE/L)	1.40 (0.75–1.82)	1.12 (0.57–1.83)	0.90
**FT4** (9.5–22.0 pmol/L)	16.6 (15.2–18.1)	17.8 (15.3–19.1)	0.45
**Creatinine** (45–90 umol/L)	60.5 (52.8–63.8)	69.5 (54.5–78.3)	0.24
**Cholesterol** (3.3–6.9 mmol/L)	5.6 (5.1–5.9)	4.7 (4.2–6.2)	0.23
**LDL-cholesterol** (1.8–5.7 mmol/L)	3.6 (3.2–3.9)	2.9 (2.5–3.9)	0.13
**HDL-cholesterol** (1.0–2.7 mmol/L)	1.5 (1.2–2.4)	1.5 (1.2–1.7)	0.68

Reference values are shown in parentheses. P-values are based on Mann-Whitney U test. PHPT, primary hyperparathyroidism; Control, patients operated for benign thyroid disease without known parathyroid or inflammatory disease; IQR, interquartile range; BMI, body-mass index; iCa, ionised calcium; APL, alkaline phosphatase; TSH, thyroid stimulating hormone; FT4, free thyroxin; LDL-cholesterol, low-density lipoprotein cholesterol; HDL-cholesterol, high-density lipoprotein cholesterol.

To adjust for the influence of age and sex on our results we selected a group of patients and controls including only females aged 27–65 years. In the age- and gender-adjusted group the average age for patients (n = 9) was 52.8 years (range 28–64) and 48.4 years (range 30–60) for controls (n = 9) (p = 0.4). In the subgroup there were not statistically significant differences in phosphate (p = 0.2) and ALP (p = 0.067). LDL-cholesterol was significantly higher in patients (3.6±0.44 mmol/L) than in controls (2.8±0.63 mmol/L) (p = 0.022). Levels of TSH, FT4, creatinine, cholesterol and HDL-cholesterol were not different between the groups.

### Shift in global gene expression in PHPT patients

**Figure 1:**
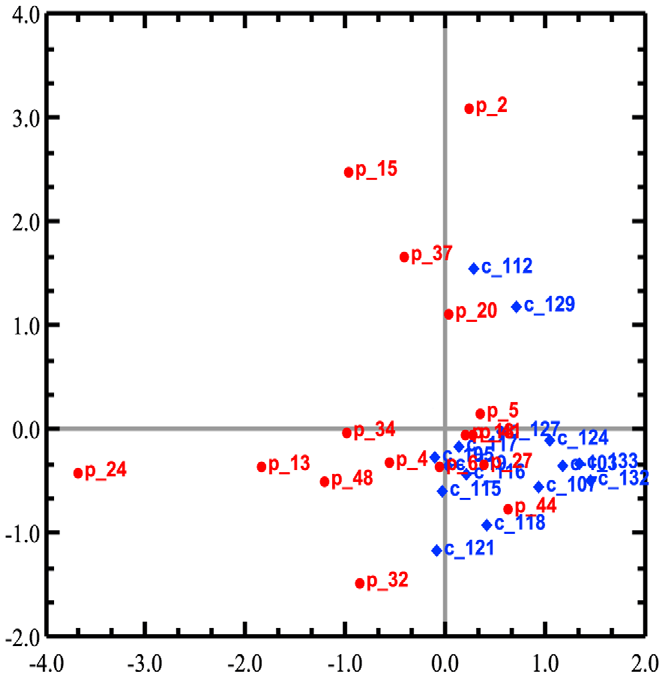
Correspondence analysis showing projection of samples. Patients with primary hyperparathyroidism are shown with red dots and the control group are shown with blue squares. The first principal component shows the largest variance in the dataset with 8.83% and the second principal component represents the second largest variance with 6.69%. Patients with primary hyperparathyroidism are separated from the control group along both axes.

Microarray analysis revealed a difference in the adipose tissue gene expression in patients with PHPT compared to controls. Correspondence analysis showed that the two groups were separated by distinct gene expression patterns, where the first principal component represented 8.83% of the total variance and the second principal component 6.69% (Fig. 1). Using Significance Analysis of Microarray (SAM), we found 608 differentially expressed genes with q-value<0.05, thereof 347 up-regulated and 261 down-regulated genes in PHPT patients compared to the control group. Several of the most up-regulated genes have previously been implicated in inflammatory diseases whereas many of the down-regulated genes play roles in lipid and carbohydrate metabolism (Table 3). Analysing the data including only females aged 27–65 years we found 162 differentially expressed genes with q-value<0.05. Of these, 113 genes were up-regulated and 49 genes were down-regulated. Correspondence Analysis of this age-matched subgroup showed equally marked differences in gene expression, with first and second principle component variance of 11.38% and 9.851%, respectively.

**Table 3:** Top 10 up-regulated and down-regulated genes in patients with PHPT compared to controls, based on Significance Analysis of Microarrays (SAM-analysis) (q-value<0.05).

		Signal Intensity		FC
Gene	Definition	PHPT	Ctr	PHPT/Ctr
S100A9	S100 calcium binding protein A9 (calgranulin B)	2429	699	3,13
S100A8	S100 calcium binding protein A8 (calgranulin A)	2367	660	2,97
MMP9	matrix metallopeptidase 9	647	229	2,55
CCL8	chemokine (C-C motif) ligand 8	2187	941	2,31
CSF3R	colony stimulating factor 3 receptor (granulocyte), transcript variant 4	653	290	2,25
TYROBP	tyrosine kinase binding protein, transcript variant 1	1917	693	2,20
ALOX5AP	arachidonate 5-lipoxygenase-activating protein	2476	940	2,16
CCL13	chemokine (C-C motif) ligand 13	824	387	2,16
THBS1	thrombospondin 1	2133	1068	2,13
CD163	CD163 molecule, transcript variant 2	1295	450	2,11
FADS1	fatty acid desaturase 1	627	1461	−2,18
CD300LG	CD300 molecule-like family member g	6838	9423	−1,76
KIF25	kinesin family member 25, transcript variant 1	394	598	−1,74
ALDOC	aldolase C, fructose-bisphosphate	6331	8326	−1,72
SCD	stearoyl-CoA desaturase (delta-9-desaturase)	18728	27771	−1,68
CA4	carbonic anhydrase IV	2516	3334	−1,66
THBS4	thrombospondin 4	2738	3779	−1,66
PPP1R1B	protein phosphatase 1, regulatory (inhibitor) subunit 1B, transcript variant 2	2320	3908	−1,66
GPIHBP1	glycosylphosphatidylinositol anchored high density lipoprotein binding protein 1	2882	3732	−1,65
ATP1A2	ATPase, Na+/K+ transporting, alpha 2 (+) polypeptide	1107	1722	−1,60

PHPT, primary hyperparathyroidism; Ctr, patients operated for benign thyroid disease without known parathyroid or inflammatory disease; FC, fold change (based on log-transformed data).

### Increased inflammatory response and reduction of metabolic processes

**Figure 2:**
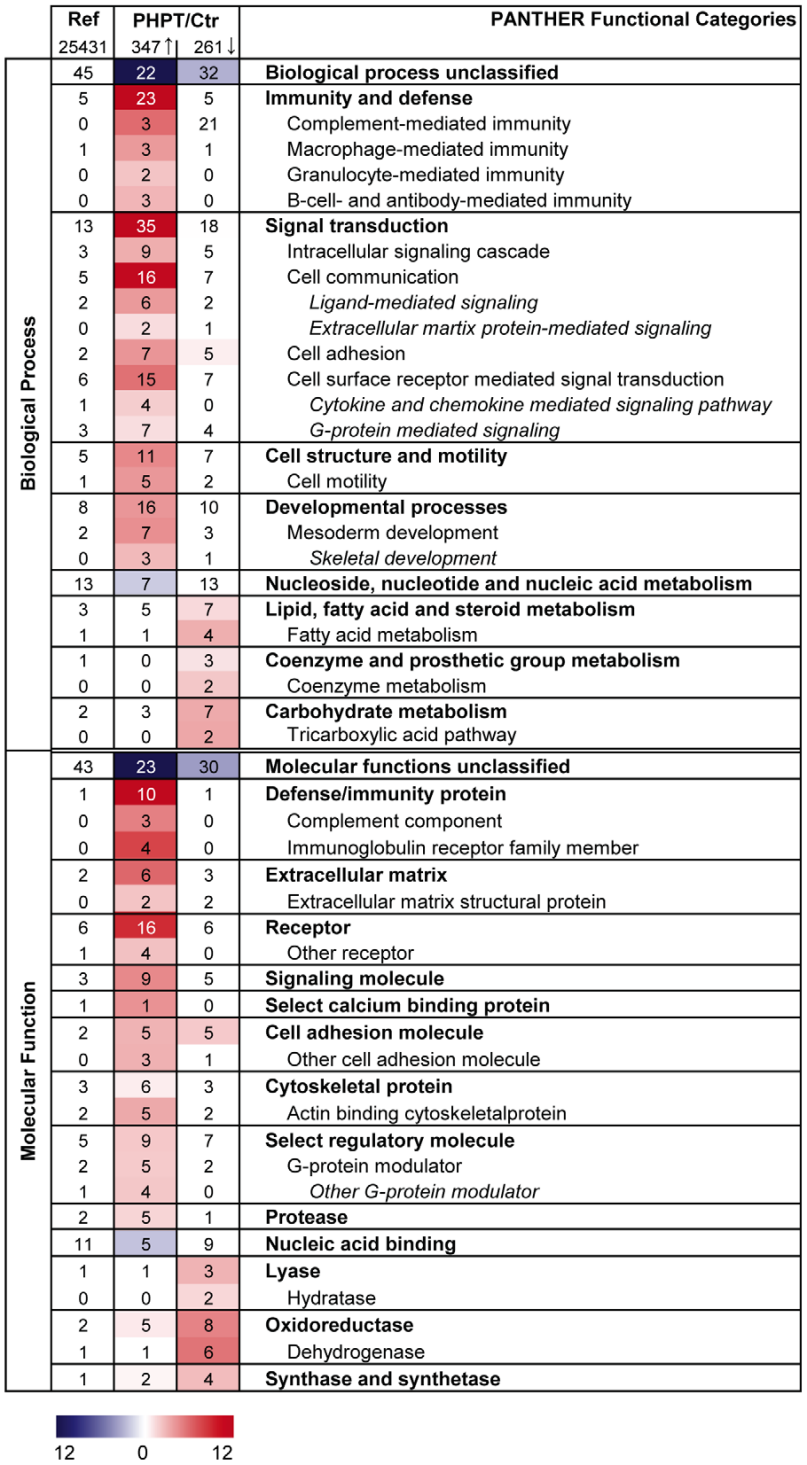
Functional categorization of differentially expressed genes (Biological Process and Molecular Function). Gene expression in subcutaneous adipose tissue in patients with primary hyperparathyroidism was compared to a control group. Over-represented Biological Processes categories and Molecular Function categories among the differentially expressed genes (q-value<0.05) were found using PANTHER. Bonferroni correction for multiple testing was done and a p-value<0.01 was used as inclusion criterion for categories. The colour intensity displays the statistical significance (−log p-value) of over- and under-represented PANTHER functional categories. Numbers in the table presents the percentage of genes mapping to a given category, e.g. 23% of the 347 up-regulated genes belonged to the Biological Process category Immunity and defense. The overall distribution of a term among all human NCBI genes (25,431) are stated in the first column, e.g. 5% of the genes are expected to map to the Biological Process category Immunity and defense, hence this category is significantly over-represented among the up-regulated genes in patients with PHPT compared to controls. Ref, Reference (based on all human NCBI genes); PHPT patients with primary hyperparathyroidism; Ctr, patients operated for benign thyroid disease without known parathyroid or inflammatory disease; Arrow up, up-regulated/higher expressed genes in patients with PHTP compared to controls; Arrow down, down-regulated/less expressed genes in patients with PHPT compared to controls.

To gain further insight into the potential functions of the differentially expressed genes, we analysed their ontology based on PANTHER functional categories (Fig. 2). Analysis of the up-regulated genes showed that the Biological Process categories Immunity and defense (e.g. S100 calcium binding protein A9 (*S100A9*, Entrez gene 6280), S100 calcium binding protein A8 (*S100A8*, Entrez gene 6279), CD14 molecule (*CD14*, Entrez gene 929)) and Signal transduction (e.g. integrin, beta2/CD18 (*ITGB2*, Entrez gene 3689), macrophage receptor with collagenous structure (*MARCO*, Entrez gene 8685)) were strongly over-represented in the PHPT patients compared to controls. Analysing the down-regulated genes in PHPT patients we found an over-representation of the Biological Process categories Lipid, fatty acid and steroid metabolism (e.g. fatty acid synthase (*FASN*, Entrez gene 2194), stearoyl-CoA desaturase (*SCD*, Entrez gene 6319)), Coenzyme and prosthetic group metabolism (e.g. acetyl-CoA carboxylase alpha (*ACACA*, Entrez gene 31), enoyl Ca-A hydratase domain containing 1 (*ECHDC1*, Entrez gene 55862)), and Carbohydrate metabolism (e.g. aldolase C, fructose-bisphosphate (*ALDOC*, Entrez gene 230), citrate synthase (*CS*, Entrez gene 1431)). Over-represented Molecular Function categories for the up-regulated genes included Defense/immunity protein (Complement component (e.g. CD55 molecule (*CD55*, Entrez gene 1604), complement factor B (*CFB*, Entrez gene 629)), Immunoglobulin receptor family member (e.g. leukocyte immunoglobuline-like receptor, subfamily B, member 5 (*LILRB5*, Entrez gene 10990), TYRO protein tyrosine kinase binding protein (*TYROBP*, Entrez gene 7305)), Extracellular Matrix (e.g. matrix metallopeptidase 9 (*MMP9*, Entrez gene 4318), collagen, type VIII, alpha 2 (*COL8A2*, Entrez gene 1296)), and Receptors (e.g. *CD14*, folate receptor 2 (*FOLR2*, Entrez gene 2350), colony stimulating factor 1 receptor (*CSF1R*, Entrez gene 1436)). For the down-regulated genes there was an over-representation of the Molecular Function groups Lyase (e.g. carbonic anhydrase (*CA4*, Entrez gene 762), malic enzyme 1 (*ME1*, Entrez gene 4199)) and Oxidoreductase (e.g. *SCD*, Entrez gene 6319, *FASN*, Entrez gene 2194).

In principle the same categories of genes were differentially expressed in the age-matched group of females aged 27–65 years as in the entire dataset. Differentially up-regulated genes were represented in the Biological Process groups Immunity and defense and Signal transduction, and several of the up-regulated genes in the whole group were also found significantly up-regulated in the subgroup (e.g. *S100A9*, *S100A8*, *MMP9*, *CD14* and *LILRB5*). As for the main group, the down-regulated genes in the age-matched group were dominated by genes mapped to metabolic processes, including lipid metabolism. Genes that were differentially expressed in the main group showed similar mean fold changes in the age-matched subgroup, though many of the q-values for differentially regulated genes in the subgroup were above 0.05. Possibly, the lack of significance for specific genes in the age-matched subgroup may have been due to the reduced sample size.

### Validation of results by qPCR

The expression levels of nine selected genes of interest were validated by real time qPCR (Table 4). The five up-regulated genes (chemokine ligand 2 (*CCL2*, Entrez gene 6347), *CD14*, *FOLR2*, *IL8* and *MMP9*) were selected as representative genes of inflammatory processes and the three down-regulated genes (fatty acid desaturase 1 (*FADS1*, Entrez gene 3992), *FASN* and *SCD*) were selected as representative genes of fatty acid and steroid metabolism. In addition, adiponectin (*ADIPOQ*, Entrez gene 9370) was measured to verify a gene that was unchanged between the patient and control group. Adiponectin is an adipokine that is specifically expressed in and released from mature fat cells, and that negatively correlates with BMI, insulin resistance, inflammation, and atherosclerosis [22]. The qPCR results were highly consistent with the microarray results, showing significant differences in expression for these genes between patients and controls, except for adiponectin that was unchanged between the groups (Table 4).

**Table 4:** Validation of selected genes by qPCR.

	Illumina, Signal Intensity			qPCR, Target gene/TBP		
	PHPT(n = 16)	Control(n = 15)	q-value	PHPT(n = 16)	Control(n = 15)	P-value
	Median	Median		Median (range)	Median (range)	
ADIPOQ	2512	3182	0.191	1.69 (0.66–2.59)	1.75 (1.29–2.88)	0.252
CCL2	1404	638	0.066	0.54 (0.10–12.89)	0.19 (0.06–0.92)	0.016
CD14	2092	909	<0.001	1.00 (0.25–3.22)	0.35 (0.18–0.72)	<0.001
FADS1	627	1461	0.029	0.45 (0.05–2.29)	1.18 (0.16–3.96)	0.040
FASN	19111	28686	0.028	2.35 (0.61–9.21)	4.58 (1.33–8.0)	0.016
FOLR2	970	423	0.054	0.47 (0.09–1.29)	0.15 (0.04–0.56)	0.001
IL8	307	190	0.109	1.02 (0.01–84.96)	0.08 (0.01–5.25)	0.022
MMP9	647	229	<0.001	3.33 (0.22–11.54)	0.53 (0.06–2.51)	0.001
SCD	18728	27771	0.028	0.35 (0.05–1.75)	0.70 (0.19–2.13)	0.030

Signal intensity measured by Illumina and median values of target genes relative to the control gene TATA-binding protein (TBP) is shown. P-values are based on Mann-Whitney U test. PHPT, primary hyperparathyroidism; Control, patients operated for benign thyroid disease without known parathyroid or inflammatory disease; q-value, adjusted p-values found using an optimised false discovery rate (FDR) approach.

### Analysis of transcription factor binding sites

To elucidate the transcriptional networks altered in the PHPT group, we used the web-based program oPOSSUM to identify frequent transcription factor binding sites in the differentially expressed genes. Analysis of up-regulated genes showed a significant enrichment of binding sites for the ETS class of transcription factors (Spi-B transcription factor (Spi1/PU.1 related), GA binding protein transcription factor (GABPA), ETS domain-containing protein Elk-1 (ELK1)), the basic leucine zipper domaine (bZIP) transcription factor activator protein 1 (AP-1/FOS), and the nuclear receptor RAR-related orphan receptor A (RORA) (Z-score>5, Fischer score <0.05) (Table 5). For the down-regulated genes, there was a significant enrichment of binding sites for the nuclear receptors estrogen receptor alpha (ESR1) and hepatocyte nuclear factor 4, alpha (HNF4A), members of the CTH2 zinc finger family (Myeloid zinc finger 1 (MZF1), Sp1 transcription factor (SP1), ras responsive element binding protein 1 (RREB1)), and the REL transcription factor NF-kappaB (Table 5). The microarray analysis showed that the mRNA levels of the transcription factors themselves were not significantly altered in PHPT patients compared to controls.

**Table 5:** Enrichment of transcription factor binding sites in the differentially expressed genes.

TF	TF Class	TF Supergroup	BG hits	BG non-hits	TG hits	TG non-hits	Z-score	Fisher score
*TF binding site in upregulated genes*								
SPIB	ETS	vertebrate	13162	1988	274	28	15.02	0.026
GABPA	ETS	vertebrate	5381	9769	122	180	7.85	0.046
RORA_1	Nuclear receptor	vertebrate	7024	8126	161	141	5.43	0.010
FOS	bZIP	vertebrate	9677	5473	208	94	5.22	0.041
ELK1	ETS	vertebrate	10697	4453	229	73	5.02	0.026
*TF binding sites in down-regulated genes*								
ESR1	Nuclear receptor	vertebrate	439	14711	13	226	7.79	0.025
ELK1	ETS	vertebrate	10697	4453	189	50	6.91	0.002
MZF1_1-4	Zn-finger, C2H2	vertebrate	13090	2060	216	23	6.90	0.041
SP1	Zn-finger, C2H2	vertebrate	9192	5958	170	69	6.36	0.001
NF-kappaB	REL	vertebrate	5960	9190	111	128	6.19	0.016
HNF4A	Nuclear receptor	vertebrate	5541	9609	112	127	5.92	0.001
RREB1	Zn-finger, C2H2	vertebrate	817	14333	20	219	5.69	0.037

TF, transcription factor; BG, background gene (expected randomly); TG, target gene.

## Discussion

Patients with PHPT are at increased risk of CVD, which may be due to a chronic low-grade inflammation. In the present study we investigated the gene expression profile of PHPT patients in subcutaneous adipose tissue from the neck. Our results indicate that patients with PHPT have inflammatory and metabolic changes in their adipose tissue.

Pro-inflammatory stimuli alter the expression of adhesion molecules on the endothelium, mediating endothelial attachment of circulating lymphocytes and monocytes and initiating early steps of atherosclerotic lesions [23]. It has previously been shown that the subcutaneous adipose tissue in morbidly obese bariatric patients expresses high levels of inflammatory genes, particularly in stromal vascular cells [15], [16]. Adipose tissue releases several of these inflammatory factors in obese subjects, which may contribute to elevated blood levels and diseases pathogenesis. Thus, it is possible that the inflammatory changes we have observed in adipose tissue of PHPT patients may result in increased circulating levels of pro-inflammatory factors, thereby increasing the risk of CVD.

*S100A8* and *S100A9* were the most up-regulated genes in the adipose tissue of PHPT patients compared to controls. These genes belong to a subgroup of the S100 family termed calgranulins, which are highly expressed in monocytes. Calgranulins mediate the induction of neutrophil chemotaxis and adhesion and have an important role in tissue inflammation [24]. Elevated levels of calgranulin are found in a wide range of acute and chronic inflammatory diseases such as rheumatoid arthritis, inflammatory bowl disease and asthma as well as in cancer [25]. It has been shown that calcium-mediated signalling is necessary for the release of S100A8/A9 [26], suggesting that their expression and possible release from adipose tissue may be increased due to elevated calcium levels in PHPT patients.

Several genes encoding the complement cascade were up-regulated in PHPT patients, including complement component 1 (*C1*) and the s-, q- and r- subcomponents of C1. The complement cascade comprises more than 30 proteins produced by various cell types, mainly hepatocytes but also monocytes and macrophages in various tissues. Activation of the complement cascade is often antibody-mediated, although antibody-independent mechanisms can act as initiators. Cleavage of C1 into C1Q, C1R and C1S further activates the cascade. This complement activation leads to production of biologically active molecules contributing to inflammation [27].

In our study *MMP9* was one of the most up-regulated genes in adipose tissue in PHPT patients compared to controls. Matrix metallopeptidases (MMPs) are a family of zinc-dependent endopeptidases involved in the degradation and reorganisation of extracellular matrix [28]. Elevated circulating levels of MMP-9 may play a role in the development of hypertension [29] and increased risk of death by CVD [30]. Moreover, MMP-9 has been implicated in atherosclerosis and atherosclerotic plaque stains positive for MMP-9 by immunhistochemistry [31]. In one study of 473 subjects, blood levels of MMP-9 were associated with grade of atherosclerosis in the femoral artery [32]. The increased expression of *MMP9* in the adipose tissue of PHPT patients may potentially contribute to the elevated risk of CVD.

An altered expression of monocyte/macrophage-related genes appears to be a hallmark of adipose tissue inflammation. Several studies have demonstrated an increased infiltration of pro-inflammatory macrophages in adipose tissue in obese patients, which may largely underlie the pathogenic potential of adipose tissue [15], [33]. Interestingly, our results indicate an increased macrophage activity in the adipose tissue of PHPT patients. Macrophage related genes that were up-regulated in PHPT patients included *CCL2 /MCP-1* (monocyte chemoattractant protein 1), *FOLR2* and *CD14*. CCL-2 acts as an important chemotactic substance that induces infiltration of monocytes into adipose tissue [34]. *CD14* is expressed on monocytes/macrophages, and activated macrophages also express an increased level of the FOLR2 [35]. The analysis of transcription factor binding sites present in the differentially expressed genes suggested that many of the up-regulated genes in PHPT might be targets of the ETS transcription factors, which have an important role in the regulation of inflammation [36]. Although mRNA levels of the transcription factor themselves are not up-regulated in PHPT patients compared to controls, the increase in genes with promoters containing binding sites for certain transcription factors possibly indicates an altered regulation by these factors. The ETS factors SpiB (PU.1 related) and PU.1 bind to almost identical ETS binding sites [37]. PU.1 may play an important role in the macrophage-related signalling cascades [38]. Binding sites for the cFOS/AP-1 transcription factor were also increased in our patient group. It has been shown that the engagement of cFOS to binding sites in macrophages up-regulates the expression of pro-inflammatory genes [39]. Together, our findings suggest that macrophage activation and infiltration contributed to the adipose tissue inflammation in the PHPT patients.

Along with the increased inflammation, our results indicate that metabolic processes are down-regulated in the adipose tissue of PHPT patients. Both anabolic and catabolic pathways of lipid metabolism seemed to be influenced. Our data suggest that adipose tissue expression of genes that are important for normal metabolic functions (e.g. *SCD*, *FASN* and *FADS1*) may be reduced in patients with PHPT. Genes encoding lipogenic enzymes such as *FASN* and *ACACA* are regulated by the transcription factors sterol regulatory element binding proteins (*SREBP*) [40]. However, mRNA levels of these transcription factors were not significantly changed in adipose tissue of patients with PHPT compared to the control group. It is worth noting that some of the metabolic genes that were down-regulated in the main group have been linked with altered insulin sensitivity and risk of CVD. Mice with a disruption in SCD-1 (stearoyl-CoA desaturase 1) have reduced adiposity, resistance to diet-induced weight gain, reduced hepatic steatosis, and increased insulin sensitivity [41]. Despite the metabolically beneficial effects, these mice developed atherosclerosis, possibly due to a macrophage inflammatory response in the artery wall [42]. In humans an increased SCD activity in adipose tissue was found to correlate with enhanced insulin sensitivity [43]. Reduced mRNA levels of *FASN* in human visceral adipose tissue were shown to correlate with higher BMI and increased metabolic dysfunction, as measured by elevated values of HbA1c, glucose levels, triglyceride and homeostasis model assessment (HOMA-IR) [44]. Together, the observations suggest that down-regulation of metabolic genes in PHPT patients may confer, or at least reflect, metabolic dysregulation.

The mechanisms that promote the altered gene expression profile in PHPT patients may involve a combined effect of elevated levels of PTH and calcium. Our gene expression data showed that adipose tissue expresses the PTH receptor, suggesting that PTH may directly induce inflammatory genes and metabolic changes in adipose tissue. A direct action of PTH on 3T3-L1 adipocytes showed a dose-dependent decrease in insulin-stimulated glucose uptake [45]. Stimulation of osteoblasts with PTH leads to an up-regulation of inflammatory proteins including interleukins [46] and MMP-9 [47]. Microarray analyses performed on parathyroid gland tissue, cultured in hypo- or hypercalcemic medium, revealed a number of genes that were consistently up-regulated or down-regulated [48]. Some of these calcium-induced genes, such as *CCL8*, were similarly affected in patients with PHPT in the present study. *MMP9* and *CFB* were down-regulated in the parathyroid gland tissue cultured in hypercalcemic medium, while these genes were up-regulated in the adipose tissue of patients with PHPT. This could be due to a suppression of PTH in the hypercalcemic cultured tissue, rather than an effect of the elevated calcium level [48]. Furthermore, the inflammatory and metabolic responses in adipose tissue of PHPT patients may have been, at least in part, secondary to the influence of PTH and calcium on other tissues.

A potential confounder in our study is that the patient group was not perfectly age-matched with the control group. Inflammation and metabolic changes could possibly be influenced by age. However, when analysing the microarray and qPCR data on age-matched subgroups we found the same patterns of differential gene regulation. Another limitation of the study is that circulating inflammatory markers or biochemical parameters indicating insulin resistance were not available. This could have given additional information concerning the changes observed in this study. It should also be noted that the control group consisted of patients operated for benign thyroid diseases. For ethical reasons these patients were the healthiest group possible to obtain as controls for our study.

Our findings highlight potentially important non-skeletal effects of elevated PTH levels in patients with PHPT. In recent years the importance of increased cardiovascular risk factors in these patients has been discussed. Our study shows highly significant alterations in gene expression in adipose tissue of PHPT patients compared to controls in regards to inflammatory and metabolic processes. The data suggest an increase in monocyte/macrophage activation in the adipose tissue. Elevated PTH and calcium may directly mediate the alterations in adipose tissue gene expression, which may in turn promote the release of pathogenic factors. Our data shed new light on inflammatory and metabolic alterations in adipose tissue in patients with PHPT that are independent of BMI, and which may confer increased risk of CVD.
